# The secondary structure of apolipoprotein A-I on 9.6-nm reconstituted high-density lipoprotein determined by EPR spectroscopy

**DOI:** 10.1111/febs.12334

**Published:** 2013-06-10

**Authors:** Michael N Oda, Madhu S Budamagunta, Mark S Borja, Jitka Petrlova, John C Voss, Jens O Lagerstedt

**Affiliations:** 1 Children's Hospital Oakland Research InstituteCA, USA; 2 Department of Biochemistry and Molecular Medicine, University of CaliforniaDavis, CA, USA; 3 Department of Experimental Medical Science, Lund UniversitySweden

**Keywords:** apolipoprotein A-I (ApoA-I), cardiovascular, cholesterol, EPR spectroscopy, high-density lipoprotein (HDL)

## Abstract

Apolipoprotein A-I (ApoA-I) is the major protein component of high-density lipoprotein (HDL), and is critical for maintenance of cholesterol homeostasis. During reverse cholesterol transport, HDL transitions between an array of subclasses, differing in size and composition. This process requires ApoA-I to adapt to changes in the shape of the HDL particle, transiting from an apolipoprotein to a myriad of HDL subclass-specific conformations. Changes in ApoA-I structure cause alterations in HDL-specific enzyme and receptor-binding properties, and thereby direct the HDL particle through the reverse cholesterol transport pathway. In this study, we used site-directed spin label spectroscopy to examine the conformational details of the ApoA-I central domain on HDL. The motional dynamics and accessibility to hydrophobic/hydrophilic relaxation agents of ApoA-I residues 99–163 on 9.6-nm reconstituted HDL was analyzed by EPR. In previous analyses, we examined residues 6–98 and 164–238 (of ApoA-I's 243 residues), and combining these findings with the current results, we have generated a full-length map of the backbone structure of reconstituted HDL-associated ApoA-I. Remarkably, given that the majority of ApoA-I's length is composed of amphipathic helices, we have identified nonhelical residues, specifically the presence of a β-strand (residues 149–157). The significance of these nonhelical residues is discussed, along with the other features, in the context of ApoA-I function in contrast to recent models derived by other methods.

## Introduction

Apolipoprotein A-I (ApoA-I) is the major protein component of high-density lipoprotein (HDL) and a key mediator of plasma cholesterol transport and cellular cholesterol homeostasis 1. During reverse cholesterol transport, ApoA-I interacts with cellular receptors/transporters such as ATP-binding cassette A1 (ABCA1), ATP-binding cassette G1 and scavenger receptor BI, which are necessary for efficient cholesterol and phospholipid efflux and delivery. In addition to these, several soluble plasma factors, including lecithin cholesterol-acyl transferase (LCAT), cholesterylester transfer protein, and an array of lipoprotein lipases, contribute to the metabolism of HDL, which include transition between lipid-free/poor ApoA-I, discoidal HDL, and spherical HDL 2. The adaptation of ApoA-I's structural organization on HDL occurs in response to both particle geometry and composition, and in turn leads to changes in HDL affinity for receptors and plasma factors. Thus, a thorough understanding of ApoA-I's structure and response to changes in HDL subclass are critical for fully understanding HDL function and its regulation.

Determining ApoA-I's structure has proven difficult for both the lipid-free state and for the HDL-associated form of the protein. Much of the challenge is attributable to ApoA-I's flexibility and plasticity, in addition to its propensity for self-association. To overcome these issues, we have employed EPR spectroscopy, which is ideal for examining proteins with a dynamic structure and is unaffected by the target protein's oligomerization state. We have determined the secondary structure of the central domain of ApoA-I on 9.6-nm reconstituted HDL (rHDL). Together with our previous analysis of the N-terminal and C-terminal domains of ApoA-I 3–4, the secondary structure of the central domain (residues 99–163) provides a complete map of ApoA-I's secondary structure on 9.6-nm rHDL particles.

## Results and Discussion

### Side chain mobility of residues in the central domain of ApoA-I on 9.6-nm rHDL

**Fig 1:**
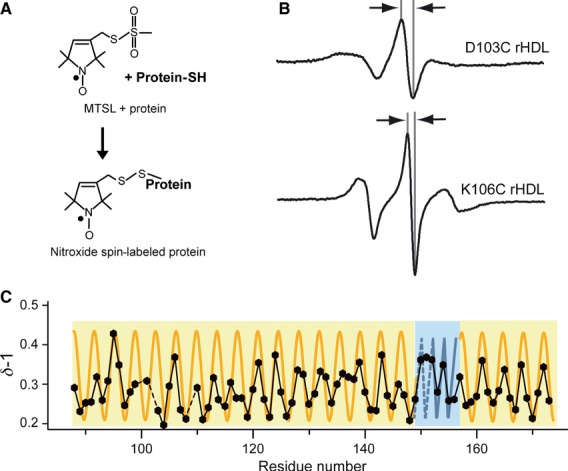
EPR spectroscopy was used to determine the structure of the central domain of ApoA-I on 9.6-nm rHDL particles. (A) ApoA-I on 9.6-nm rHDL bearing a nitroxide label (MTSL) was analyzed by EPR spectroscopy. (B) The central line width (arrows) of the resulting spectra is representative of the labeled amino acid's side chain mobility, where broader (D103C, top) and narrower (K106C, bottom) line widths indicate low and high mobility, respectively. (C) The inverse spectral line width (*δ*^−1^) plotted as a function of residue number is used to identify secondary structure elements. Solid and dashed black lines indicate consecutive and missing data points, respectively. A sinusoidal-shaped periodicity of 3.6 residues per cycle indicates an α-helical structure (orange), whereas an alternating periodicity indicates a β-strand structure (blue). Data for residues 88–173 are plotted, wherein residues 99–163 are from the current study.

**Fig 2:**
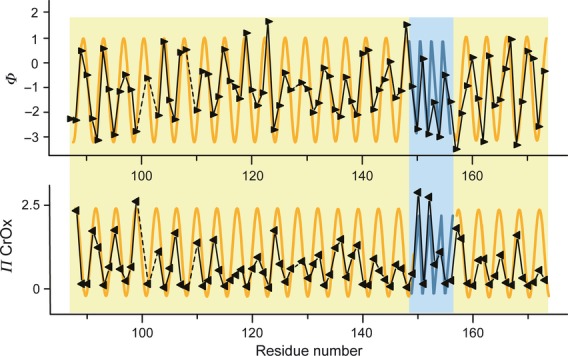
The accessibility of diffusible spin relaxers provide a measure of the hydrophobicity of the spin-labeled side chain environment. Top: The contrast function (Φ), which is the logarithmic value of the ratio between the hydrophobic (O_2_) and the hydrophilic (CrOx) paramagnetic relaxers, is a relative measure of the hydrophobicity of the local microenvironment of the labeled side chain, and is plotted as a function of residue number. Bottom: the accessibility parameter of the hydrophilic relaxer (*Π*_CrOx_), a measure of the relative hydrophilicity of the local microenvironment of the labeled side chain, is plotted as a function of residue number. Larger contrast function values and lower accessibility values of the hydrophilic relaxer indicate greater hydrophobicity. Solid and dashed black lines indicate consecutive and missing data points, respectively. A sinusoidal-shaped periodicity of 3.6 residues per cycle indicates an α-helical structure (orange), whereas an alternating periodicity indicates a β-strand structure (blue). Data for residues 88–173 are plotted, wherein residues 99–163 are from the current study.

Sixty-two single-Cys ApoA-I variants with substitutions within residues 99–163 were covalently nitroxide spin-labeled by the use of thiol chemistry, reconstituted on 9.6-nm rHDL, and analyzed by EPR spectroscopy (Fig. 1). The shapes of the spectra provide direct information on the degree of motional freedom of the labeled side chain (e.g. residues 103 and 106; Fig. 1B), wherein sharp and broadened spectra indicate high and low motional freedom, respectively. The mobility parameter *δ*^−1^, which is the inverse of the central line width, provides a relative measure of side chain mobility 5, and was used here to determine ApoA-I's central domain secondary structure (Fig. 1C). Mutants with Cys substitutions at residues 100, 102 and 109 were excluded from analyses, owing to low protein solubility. Figures 1C and 2 include data from an additional 10 residues upstream and downstream (3 and 4, respectively) of the central domain residues (99–163) to demonstrate that, although we have defined residues 99–163 as the central domain, this is a numerical assignment, and does not define a conformational domain. Within this domain, there are four regions (103–116, 118–136, 138–148, and 160–173) that show a pattern of *δ*^−1^ with a periodicity indicative of a typical α-helix (3.6; see orange sinusoidal curve in Fig. 1C). However, residues 149–159 do not follow this pattern, but instead show an alternating periodicity indicative of β-strand secondary structure. The exception to this is Arg151, whose mobility (*δ*^−1^ = 0.37) does not conform to the alternating pattern that one would expect for a β-strand. Given the relatively high mobility of its neighbors (positions 150 and 152), one would expect *δ*^−1^ for position 151 to fall within the immobile range of this series (≤ 0.25), which it does not. Interestingly, the chromium oxalate (CrOx) accessibility of position 151 is significantly lower than that of its neighbors, which conforms to an alternating periodicity of a β-strand. There is more than one possible explanation for why position 151 does not show the degree of mobility expected of a residue on a β-strand. For instance, a stable β-strand may only transiently exist at position 151, narrowing the EPR spectrum via rapid backbone motions. Also, the pattern of mobility is obscured for a span of residues of fixed secondary structure when there are discontinuities in surface exposure, as mobility is a function not only of the extent of each residue's contact with other molecules, but also of its degree of solvent exposure. In addition to the mobility discrepancy at position 151, the mobility of residues 117 and 137 is ambiguous, and it is difficult to discern by mobility alone whether these residues reside within a contiguous helix (residues 103–148). As with residue 151, this ambiguity is resolved by determining the pattern of residue-specific molecular accessibility/degree of solvent exposure (see below).

### Solvent accessibility of the central domain of ApoA-I on 9.6-nm rHDL

The molecular accessibility of ApoA-I central domain residues on 9.6-nm rHDL particles was determined by observing the relative susceptibility of nitroxide label to the presence of hydrophilic (CrOx) or hydrophobic (O_2_) paramagnetic relaxation agents. The degree of attenuation of spectral intensity attributable to the collision frequency of a diffusible relaxer and the nitroxide spin label is a quantitative measure of the solvent accessibility of the nitroxide-modified side chain. In Fig. 2, the accessibility parameter (*Π*) of CrOx is plotted as a function of residue. Molecular accessibility was determined for all residues between positions 99 and 163, with the exception of residues 100, 102, 109, and 128.

**Fig 3:**
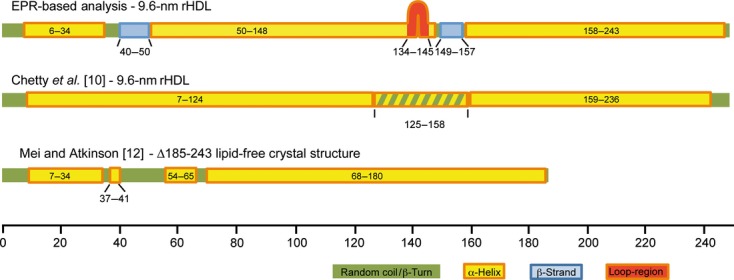
Comparison of secondary structure models of ApoA-I. The secondary structure maps of ApoA-I as determined by EPR spectroscopy (top), HX MS analysis (middle 10) and X-ray crystallography (residues 1–184; bottom 12–13) are shown. The position of the loop-like element (residues 134–145) defined in the ‘looped belt’ model 7 is indicated.

To identify the residues whose side chains are partitioned into the lipid environment of the HDL particle, the relative collision frequency of O_2_ was compared with that of CrOx (polarity contrast function, Φ; Fig. 2, upper panel). For residues in an exposed hydrophilic location, *Π*_CrOx_ is higher in value than *Π*_O2_, resulting in negative Φ values. For residues in a hydrophobic environment, *Π*_O2_ is greater than *Π*_CrOx_, and Φ is more positive in value. This approach is specifically useful for proteins that have significant exposure to both hydrophilic and hydrophobic environments, such as lipid-bound ApoA-I protein. The plotted values of *Π*_CrOx_ and Φ for ApoA-I residues (Fig. 2) show the hallmark sinusoidal pattern of solvent accessibility that is indicative of an α-helical structure, consistent with mobility analysis observations. In addition, the data support a continuous helical structure for residues 117 and 137 (Fig. 1C). As discussed above, the apparent lack of fitting of the mobility of residues 117 and 137 to a helical pattern is probably explained by deviations in surface contact/exposure (e.g. intramolecular and/or intermolecular protein contact). The mobility of position 137 is consistent with the ‘looped belt’ model, wherein residues 134–145 form a loop-like element on 9.6-nm rHDL particles 6–7, and a majority of these residues maintain their helical facing to the lipid environment of the HDL particle (Fig. 3). Thus, the loop of the ‘looped belt’ model is not derived from a random coil loop but rather from an arced helix, with hinges at residues 133, 139, and 146 7, consistent with the molecular dynamics simulations of Segrest *et al*. 8. The altered facing of residue 137 is consistent with a deviation in protein–lipid contact that one would expect near a helix hinge. The polar accessibility parameter (*Π*_CrOx_) and contrast function (Φ) show an alternating pattern for residues 149–157, consistent with a β-strand structure.

### Secondary structure of ApoA-I on 9.6-nm rHDL–EPR versus other approaches

Combined with our previous EPR analyses of the N-terminal 3 and C-terminal 4 domains of ApoA-I, our current analysis provides a complete description of the secondary structure of ApoA-I on 9.6-nm rHDL (Fig. 3). The structure has two major helical regions, residues 50–148 and residues 158–243, that are separated by a short stretch of amino acids (residues 149–157) that form a β-strand. There is a second β-strand structure at residues 40–50, which neighbors a short random coil segment (residues 35–39) that is connected to an α-helix (residues 6–34). The average helical percentage of ApoA-I in this structure is ∼ 85%, which is similar to the helical content determined with CD spectroscopy (∼ 78%) 9.

Recently, Chetty *et al*. 10 used hydrogen–deuterium exchange (HX) combined with MS analysis to determine the solution structure of ApoA-I on rHDL. The secondary structure distribution from their analysis identified two major helical segments (residues 7–124 and 159–236) separated by a bimodal region with two structural states (helical and random coil) that interchange on the minute scale. Several features of the HX model are in agreement with the EPR-based structure. These include an extended α-helix in the C-terminal domain and also a significant portion of helical structure in the N-terminal and central domains (Fig. 3). Interestingly, part of the region described as bimodal in the HX model corresponds to a region of the central domain that EPR analysis identifies as a β-strand. Both methods support a nonhelical character for this region, although they arrive at different conclusions with respect to whether it is an organized or disorganized structure (random coil versus β-strand). In contrast to this general agreement, the random coil and β-strand structures defined by EPR in the N-terminal domain (residues 35–50) are not present in the HX model. The reason for this difference may be the concentrations of HDL employed in the two studies, or the difference in resolution of the methods. EPR and HX experiments were performed at 60 μm and 5 μm ApoA-I (or 30 μm and 2.5 μm rHDL), respectively. Although the effect of concentration on the discoidal HDL ApoA-I structure is unknown, the structure and oligomeric state of lipid-free ApoA-I are concentration dependent 11. Further studies are warranted to determine whether there is a relationship between HDL concentration and resident ApoA-I structure, as this would have an impact on our understanding of the regulation of interactions between HDL and its associated receptors/enzymes. This differential observation may also be a result of the ability of HX to identify β-strands in proteins, as this technique has a limited resolution at the peptide level, whereas EPR can distinguish structural parameters at the residue level. Nevertheless, despite these discrepancies, both methods show this region to contain nonhelical structures.

**Fig 4:**
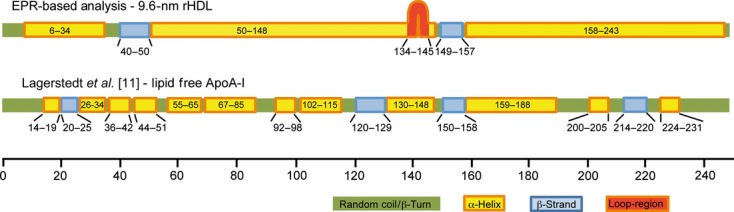
Schematic representation of secondary structure distribution of ApoA-I on 9.6-nm rHDL as compared with lipid-free ApoA-I. Secondary structure depiction from this study (residues 99–163), Lagerstedt *et al*. 3 (residues 1–98 3) and Oda *et al*. 4 (residues 163–243) of lipid-bound 9.6-nm rHDL particles (top) and lipid-free ApoA-I (bottom 11). The position of the loop-like element (residues 134–145) defined in the ‘looped belt’ model 7 is indicated.

We next compared our results with the recently published crystal structure of a fragment of ApoA-I spanning residues 1–184 12. Despite being crystallized in the absence of lipids, several features of the crystal structure are rHDL-like in conformation 13. These features include a curved elongated helical structure with a diameter of ∼ 11 nm. Interestingly, both the short helix (residues 6–34) and a random coil/β-strand region (residues 35–50) observed by EPR spectroscopy in the N-terminal domain of ApoA-I on rHDL are found in the crystal structure of the fragment (Fig. 4), supporting our results and suggesting that the recent crystal structure is more likely to be representative of a lipid-bound conformation. Interestingly, the N-terminal structure in lipid-free ApoA-I, as determined by EPR spectroscopic analysis 11, is significantly altered upon rHDL association 3.

### Transition of ApoA-I structure from the apo-state to discoidal 9.6-nm rHDL

During lipid binding and HDL maturation, ApoA-I undergoes a series of changes in conformation 14. In a previous EPR analysis of lipid-free ApoA-I, we determined that its secondary structure is divided into shorter helical regions (< 30 residues) separated by random coil or β-strand regions (Fig. 4) 11. Four β-strand segments are identified in the lipid-free solution structure. Of these, one β-strand is also present in ApoA-I on 9.6-nm rHDL (residues 149/150 to 157/158). The fact that residues 149–157 maintain a conserved β-strand structure in both lipid-free and lipid-bound ApoA-I (Fig. 4) suggests a specific role of this region in the biogenesis and maturation of HDL. This is underscored by the number of variants of ApoA-I with amino acid substitutions in, or near, this region that have reduced LCAT activation and/or low levels of plasma ApoA-I/HDL. These include the R151C Paris 15, L159R Finnish 16 and L159P Zavalla 17 variants. In addition, the central residue in this β-strand, Arg153, is completely conserved in mammals, birds, and amphibians 18, and is important for LCAT binding to HDL 19, which further emphasizes the importance of this residue and its proximal residues in HDL maturation. The importance of this region in the interaction with ABCA1, ATP-binding cassette G1, scavenger receptor BI and cholesterylester transfer protein is not clear. However, although a conformational rearrangement of the central region occurs during ABCA1-mediated lipid binding, recent data instead indicate that the N-terminal and C-terminal regions of ApoA-I interact directly with ABCA1 20–21 suggesting a minor role of the identified ApoA-I β-strand in the ABCA1 interaction.

In addition to regions that transition from β-strand to α-helix upon lipid binding, one region that is primarily helical in lipid-free ApoA-I (residues 40–50) adopts a β-strand structure on 9.6-nm rHDL. Interestingly, the solvent exposure of this region is significantly altered during the expansion of smaller discoidal HDL particles (diameter of 7.8 nm) to 9.6-nm HDL particles 3, but the β-strand is retained as a consistent element in the rHDL particle. This suggests that, unlike the other ApoA-I β-strands that transition to α-helix during HDL formation 11, residues 40–50 do not play a role in the stabilization of the lipid-free protein, but rather may be important in the stability and/or maturation of HDL. The rationale underlying this conclusion is that transient β-strand elements in lipid-free ApoA-I serve as pseudolipids, providing a nucleation center for the hydrophobic face of ApoA-I's amphipathic helices, thereby stabilizing the lipid-free ApoA-I helical bundle 11. Upon lipid binding, these β-strands transition to α-helices, eliminating the amphipathic helices' hydrophobic nucleus, and driving the helices to partition into the lipid environment of the forming HDL particle.

## Conclusion

The current analysis provides a complete and detailed description of the secondary structure of ApoA-I on 9.6-nm rHDL. From this study, we conclude that the majority of the protein on 9.6-nm rHDL adopts a helical structure (residues 6–34, 50–148, and 158–243), with the exception of residues 40–50 and 149–158, which form β-strands. The helical content in this structure is ∼ 85% and the β-strand content is ∼ 9%, both of which are similar to the values determined with CD spectroscopy (70–78% α-helical 9,20 and 10–11% β-strand 22–23. Several of the structural features identified by EPR are present in a recently proposed structure based on HX analysis 10. However, there are significant differences in the structural assignments of the two methods. On the basis of our EPR analyses, EPR provides better resolution in defining helical regions, and is able to discern β-strands from random coils. The crystal structure of truncated lipid-free ApoA-I (residues 1–184), although a lipid-free peptide, might be more representative of HDL-associated ApoA-I 13, which is analogous to the observations made of the crystal structure of an N-terminal truncation of ApoA-I (Δ1–43) 24. It is intriguing that the crystal model of residues 1–184 is similar to our EPR-derived rHDL model, most notably in that both depict residues 40–50 as nonhelical. Whereas the β-strand at residues 40–50 may play a key role in the adaptation of ApoA-I to changes in discoidal HDL size 3, we speculate that the β-strand at residues 149–158 is important for establishing the proper alignment of ApoA-I monomers in the lipid-free apo-state, which establishes the alignment of ApoA-I monomers on discoidal particles 7 that are subsequently stabilized by salt bridging 25. This hypothesis is further supported by a molecular dynamics simulation that positions residues 149–158 proximal to their counterparts on a paired ApoA-I during HDL formation 8. Furthermore, after discoidal HDL formation, the β-strand at residues 149–158 may be critical for the interaction with LCAT by presenting a unique protein-binding element, in which β-strands are commonly utilized. We will employ this structural model of ApoA-I on 9.6-nm HDL as the basis for future experimental work to investigate the relationship between ApoA-I structural properties and HDL function.

## Experimental procedures

### Materials

Thio-specific nitroxide spin label (1-oxyl-2,2,5,5-tetramethylpyrroline-3-methyl)-methanethiosulfonate) was purchased from Toronto Research Chemicals (Toronto). 1-Palmitoyl-2-oleoyl-*sn*-glycero-phosphocholine (POPC) and cholesterol were purchased from Avanti Polar Lipids (Alabaster, AL, USA).

### Production of recombinant and spin-labeled ApoA-I

Sixty-two single Cys substitutions of ApoA-I (P99C–L163C) were previously created by either primer-directed PCR mutagenesis or the megaprimer PCR method 11. The proteins were expressed, purified and reacted with a methanethiosulfonate spin label (MTSL), as previously described 4. In brief, 8 mg of protein was sequentially incubated with 100 μm Tris-(2-carboxyethyl)phosphine and 300 μm MTSL on an Ni^2+^ -chelated HiTrap column (GE Healthcare) under denaturing conditions (3 m guanidine-HCl), extensively washed with NaCl/P_i_ (20 mm phosphate, 500 mm NaCl), and eluted with imidazole. Protein purity (> 95%) was confirmed by SDS/PAGE.

### HDL reconstitution

rHDLs were prepared with the deoxycholate method 26–27. Dried POPC and free cholesterol were resuspended in NaCl/Tris (8.2 mm Tris/HCl, 150 mm NaCl, 0.1 mm EDTA), pH 8.0, with 19 mm sodium deoxycholate. The mixture was vortexed and incubated at 37 °C until it was clarified. Spin-labeled ApoA-I was added to the mixture, and incubated for 1 h at 37 °C. Deoxycholate was removed by extensive dialysis against NaCl/Tris (pH 8). Protein/lipid molar ratios (ApoA-I/free cholesterol/POPC) were 1 : 4 : 80 for 9.6-nm rHDL. Residual lipid-free protein was removed from the preparation by KBr density gradient ultracentrifugation at 50 000 ***g*** for 3 h in a Beckman Optima TLA 100.4 rotor. Homogeneous rHDL was isolated by size exclusion chromatography as previously described 28. The size and purity of lipidated discoidal complexes was confirmed by nondenaturing gradient gel electrophoresis, as previously described 29.

### EPR analysis

A JEOL X-band spectrometer fitted with a loop-gap resonator 30 was used for EPR measurements. Aliquots (5 μL) of purified rHDL (60 μm spin-labeled protein) were loaded into quartz capillaries, and spectra were collected at room temperature (20–22 °C) from a single 2-min scan with 100 G sweep width at a microwave power of 2 mW and a modulation amplitude optimized to the natural line width of the individual spectrum (0.5–1.5 G). For integration of spin counts, samples were structurally dispersed by addition of SDS to a final concentration of 2% (w/v), and the resulting EPR spectrum was double-integrated for total spin intensity. The molecular accessibility of spin-labeled side chains to the paramagnetic relaxation agents CrOx and O_2_ was determined by the use of successive power saturation scans 31–32. These measurements provide a normalized value for the collision frequency (*Π*) of the diffusible agent. The Φ parameter describes the polarity of the local environment, and is calculated from the log of the ratio between polar (*Π*_CrOx_) and apolar (*Π*_O2_) relaxers, and is especially useful in analyses of protein partitioning in lipid assemblies 33.

### Molecular modeling

chimera (UCSF) 34 was used to compare the crystal structure of the 1–184 fragment (Protein Data Bank 3R2P) 12 and a model of lipid-bound ApoA-I on 9.6-nm rHDL 3.

## Abbreviations

ABCA1: ATP-binding cassette A1
ApoA-I: apolipoprotein A-I
CrOx: chromium oxalate
HDL: high-density lipoprotein
HX: hydrogen–deuterium exchange
LCAT: lecithin cholesterol-acyl transferase
MTSL: methanethiosulfonate spin label
POPC: 1-palmitoyl-2-oleoyl-*sn*-glycero-phosphocholine
rHDL: reconstituted high-density lipoprotein

## Supporting information

Fig S1 Examples of EPR spectra of spin-labeled apoA-I proteins within the 99–163 region of the primary sequence.
