# Supplementary material for: The secondary structure of apolipoprotein A-I on 9.6-nm reconstituted high-density lipoprotein determined by EPR spectroscopy
Source: FEBS J. 2013 Jun 10;280(14):3416–24. doi: 10.1111/febs.12334 (PMC3906832; doi:10.1111/febs.12334)
Supplement: Fig S1 — Examples of EPR spectra of spin-labeled apoA-I proteins within the 99–163 region of the primary sequence. [file febs0280-3416-sd1.pdf]

the FEBS Journal
www.febsjournal.org

The secondary structure of apolipoprotein A-I on 9.6-nm reconstituted high-density lipoprotein determined by EPR spectroscopy

Michael N. Oda, Madhu S. Budamagunta, Mark S. Borja, Jitka Petrlova, John C. Voss and Jens O. Lagerstedt

DOI: 10.1111/febs.12334

Supplementary figure 1 – Oda et al.

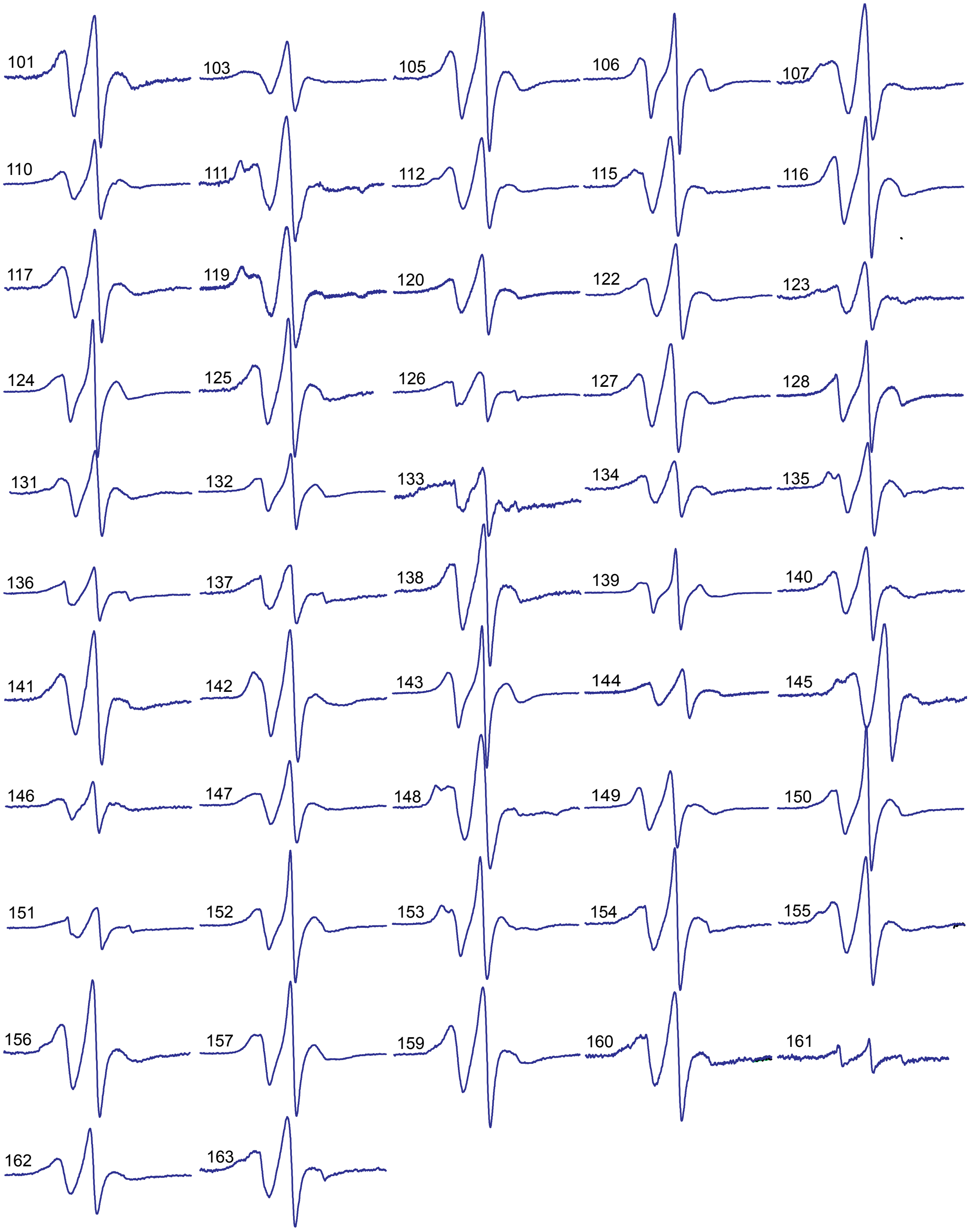

Fig S1. Examples of EPR spectra of spin-labeled apoA-I proteins within the 99-163 region of the primary sequence.
